# Exploratory and inferential analysis of gene cluster neighborhood graphs

**DOI:** 10.1186/1471-2105-10-288

**Published:** 2009-09-14

**Authors:** Theresa Scharl, Ingo Voglhuber, Friedrich Leisch

**Affiliations:** 1 Department of Statistics and Probability Theory, Vienna University of Technology, Wiedner Hauptstr. 8-10, A-1040 Vienna, Austria; 2 Department of Biotechnology, University of Natural Resources and Applied Life Sciences, Vienna, Muthgasse 18, A-1190 Vienna, Austria; 3 Department of Statistics, University of Munich, Ludwigstr. 33, D-80539 Munich, Germany

## Abstract

**Background:**

Many different cluster methods are frequently used in gene expression data analysis to find groups of co-expressed genes. However, cluster algorithms with the ability to visualize the resulting clusters are usually preferred. The visualization of gene clusters gives practitioners an understanding of the cluster structure of their data and makes it easier to interpret the cluster results.

**Results:**

In this paper recent extensions of R package **gcExplorer **are presented. **gcExplorer **is an interactive visualization toolbox for the investigation of the overall cluster structure as well as single clusters. The different visualization options including arbitrary node and panel functions are described in detail. Finally the toolbox can be used to investigate the quality of a given clustering graphically as well as theoretically by testing the association between a partition and a functional group under study.

**Conclusion:**

It is shown that **gcExplorer **is a very helpful tool for a general exploration of microarray experiments. The identification of potentially interesting gene candidates or functional groups is substantially accelerated and eased. Inferential analysis on a cluster solution is used to judge its ability to provide insight into the underlying mechanistic biology of the experiment.

## Background

Cluster analysis is frequently used in gene expression data analysis to find groups of co-expressed genes which can finally suggest functional pathways and interactions between genes. Clusters of co-expressed genes can help to discover potentially co-regulated genes or association to conditions under investigation. Usually cluster analysis provides a good initial investigation of microarray data before actually focusing on functional subgroups of interest. Genetic interactions are complex and the definition of gene clusters is often not clear. Additionally microarray data are very noisy and co-expressed genes can end up in different clusters. Therefore the set of genes may be divided into artificial subsets where relationships between clusters play an important role.

In the literature numerous methods for clustering gene expression data have been proposed. Detailed reviews of currently used methods and challenges with gene expression data are given in [1-3]. The display of cluster solutions particularly for a large number of clusters is very important in exploratory data analysis. Visualization methods are necessary in order to make cluster analysis useful for practitioners. They give an understanding of the relationships between segments of a partition and make it easier to interpret the cluster results. In hierarchical clustering dendrograms and heatmaps are routinely used (e.g., [4]). The most popular group of partitioning cluster algorithms are centroid-based cluster algorithms (e.g., K-means or Partitioning Around Medoids). Once a set of centroids has been found centroid-based cluster solutions are usually visualized by projection of the data into two dimensions (e.g., by principal component analysis). Silhouette plots [5] can be used to check whether clusters of points are well separated whereas topology representing networks [6] reveal similarity between clusters. Neighborhood graphs [7] combine these two approaches to visualize cluster structure.

In this paper recent extensions of R package **gcExplorer **[8] are presented. In the package neighborhood graphs are used for visual assessment of the cluster structure. Several node functions can be used to add further information to the graph, e.g., cluster size or cluster tightness. Additionally it is possible to use distinct graphical symbols for the representation of single clusters, e.g. line plots or boxplots. Beside the node function a panel function is implemented allowing to explore the corresponding clusters interactively in more detail by looking at arbitrary cluster plots or HTML tables of the group of genes under investigation. Further, external information about the genes like gene function or association to gene sets like Gene Ontology [9] can easily be integrated into the exploration. Finally the toolbox can be used to investigate the quality of a given clustering graphically as well as theoretically. In the functional relevance test the association between a partition and a functional group under study is tested. Further, the validity of a cluster solution under different experimental conditions is tested.

## Methods

The visualization methods discussed in this paper are designed for cluster solutions of partitioning cluster algorithms where clusters can be represented by centroids (e.g., K-means and PAM or QT-Clust [10]).

Neighborhood graphs

Neighborhood graphs [7] use the mean relative distances between points and centers as edge weights in order to measure how separated pairs of clusters are. Hence they display the distance between clusters. In the graph each node corresponds to a cluster centroid and two nodes are connected by an edge if there exists at least one point that has these two as closest and second-closest centroid.

For a given data set *X*_*N *_= {*x*_1_,..., *x*_*N*_} the distance between points *x *and *y *is given by *d*(*x, y*), e.g., the Euclidean or absolute distance. *C*_*K *_= {*c*_1_,..., *c*_*N*_} is a set of centroids and the centroid closest to *x *is denoted by



The second closest centroid to *x *is denoted by



The set of all points where *c*_*k *_is the closest centroid is given by



Now the set of all points where *c*_*i *_is the closest centroid and *c*_*j *_is second-closest is given by



For each observation *x *the shadow value *s*(*x*) is defined as



*s*(*x*) is small if *x *is close to its cluster centroid and close to 1 if it is almost equidistant between the two cluster centroids. The average s-value of all points where cluster *i *is closest and cluster *j *is second closest can be used as a proximity measure between clusters and as edge weight in the graph.



|*A*_*i*_| is used in the denominator instead of |*A*_*ij*_| to make sure that a small set *A*_*ij *_consisting only of badly clustered points with large shadow values does not induce large cluster similarity.

### Functional relevance test

Now the obtained similarity between clusters and the neighborhood graph can be used to evaluate a cluster result at hand. The cluster structure can be used to decide whether the clustering is too coarse and needs further subdivision to respect the data or if it is too fine and some clusters should be merged. On the one hand this can be accomplished by defining some threshold *t *for the shadow value *s *above which two clusters are merged. In the case of too large clusters more accurate clusters can for instance be obtained by running the algorithm again with larger *K*.

On the other hand external knowledge about the data can be used to validate a given clustering. In the case of microarray data a priori information about gene function or the association to functional groups can be used as functionally related genes are more likely to be co-expressed. Clusters with similar expression pattern are connected in the neighborhood graph. If functional group *F *is independent of the experimental setup genes classified to group *F *will be assigned to arbitrary clusters, i.e., they are assumed to be spread all over the neighborhood graph. Further, genes functionally independent of the experimental setup do not have a common expression pattern. If functional group *F *plays a role in the experiment the corresponding genes are more likely to show a typical pattern of either up- or down-regulation and there should be clusters with accumulation of such genes.

Assigning all genes in the clustered data set to some functional group *F *yields proportions *π*_1_,..., *π*_*K*_where *K *is the number of clusters or nodes and *N*_*F *_is the total number of genes in the data set assigned to group *F*. If there is no association between the functional group and the cluster solution then all proportions are the same, i.e., the differences between proportions *d*_*ij *_= 0 where



If there is an association then some *π*_*k *_will be large and others small. The test for functional relevance of a given clustering is conducted in a stepwise way.

**Step 1**: Perform a global test of the equality of proportions, i.e., test the null hypothesis that all proportions  are the same



The test procedure stops if there is no difference in proportions. But if there are significant differences in proportions each single difference has to be investigated in more detail. If the proportion of functionally related genes is the same in two clusters these two clusters are similar with respect to functional group *F *and can therefore be merged. This procedure yields separated subgraphs with common gene function within the neighborhood graph.

Without knowledge about the cluster structure and the similarities between clusters given in the neighborhood graph *G *each pair of clusters has to be tested for a significant difference in proportions, i.e., *K*(*K *- 1)/2 tests have to be conducted. Using the neighborhood structure only a fraction of all possible pairs, i.e., clusters connected by an edge have to be tested. A further reduction of tests can be achieved by taking into account only nodes where the number of functionally assigned genes is above a threshold *m*.

**Step 2**: Assess the significance of the observed differences with respect to a reference distribution by permuting the function labels. The null hypothesis is again no difference in proportions.

• Select all clusters where the number of functionally assigned genes is above the predefined threshold *m *and conduct all further calculations on the resulting subgraph *G'*.

• Calculate the difference between proportions *d*_*ij*_, *i, j *= 1,..., *K *for each edge in the subgraph.

• Permute the function labels, i.e., randomly assign  genes to functional group *F*, where  is the number of assigned genes in the subgraph *G' *with  ≤ *N*_*F*_. Compute the resulting differences in proportions , *i, j *= 1,..., *K *and keep the respective maximum



as used in [11] to form a reference distribution  where *L *is the number of permutations considered.

• Compute marginal tests whether a particular *d*_*ij *_is extreme relative to the joint distribution *M*^*l*^, i.e., compute how often the maximum of the permuted differences in proportions is larger than the observed one.

In other words, if the observed difference in proportions is very unlikely with respect to the reference distribution of the maxima *M*^*l *^the edge will be removed. In this procedure a modified neighborhood graph is formed for the cluster solution and functional group under investigation. In this modified graph two clusters are only connected if they have

1. a large similarity value *s *and

2. no significant difference in proportions of functionally related genes.

### Compare cluster results

Validation of microarray cluster results is a challenging task (e.g., [2]) as there is in general no true cluster membership. The quality of a cluster solution should be judged based on its ability to provide insight into the underlying mechanistic biology. As described in the previous section the validity of a cluster solution can be judged based on its ability to find groups of functionally related genes. Another approach is to find genes with common mechanism of regulation by searching for groups of genes that show a common response in different experiments.

For that purpose another test procedure was developed. We test how valid a given cluster solution is on a different data set taking into account the average within cluster distance *W *= (*w*_1_,..., *w*_*K*_) where



Let *X*_*N *_be the data matrix of *N *genes for a given experiment and let *M *be the vector of length *N *of the corresponding cluster memberships. Further let *Y*_*N *_be the data matrix of the same *N *genes in a different experiment. In order to test if the cluster memberships *M *found for data set *X*_*N *_are also valid in data set *Y*_*N *_the following procedure is used.

1. Compute the new cluster centroids  for data set *Y*_*N *_using the vector of cluster memberships *M*.

2. For each cluster *k *compute the average within cluster distance of data points *y*_*n *_to their assigned centroid , i.e.,



3. Permute the cluster memberships, i.e., randomly assign the genes to clusters but do not modify cluster sizes. Compute the resulting average within cluster distance  for each cluster and keep the  where *L *is the number of permutations considered.

4. Compute marginal tests for each cluster of whether a particular  is extreme relative to the joint distribution of .

For each *k *where *k *= 1,..., *K *a single test is performed with the null hypothesis



and the alternative hypothesis is



The null hypothesis is rejected if the propability of observing a smaller within cluster distance by randomly assigning genes to clusters is less than e.g. 5%. In this case there is a relationship between the investigated cluster solution on the original data set and on the new data set and genes with common expression pattern across experiments are found.

### Data

*E. coli *cultivation data were collected at the Department of Biotechnology of the University of Natural Resources and Applied Life Sciences in Vienna. Two recombinant *E. coli *processes with different induction strategies were conducted in order to evaluate the influence of the expression level of the inclusion body forming protein N^*pro*^GFPmut3.1 on the host metabolism [12]. The standard strategy with a single pulse of inducer yielding in a fully induced system was compared to a process with continuous supply of limiting amounts of inducer resulting in a partially induced system [13]. In order to analyze the cellular response to different induction strategies on the transcription level two independent DNA microarray experiments were performed. A dye-swap design was used and the cells in the non-induced state of each experiment were compared to samples past induction. The two experiments are available at ArrayExpress . The experiment with fully induced *E. coli *expression system has accession number E-MARS-16 and the experiment with partially induced system has accession number E-MARS-17. For standard low level analysis the data were preprocessed using print-tip loess normalization. Differential expression estimates were calculated using Bioconductor ([14], ) package **limma **[15]. The two data sets were filtered by selecting genes with p-value of the corresponding F-statistic smaller 0.05. Additionally, only genes expressed at a certain level (average log intensity A larger 8) and genes with clearly defined pattern (log-ratio M larger ± 1.5 at least at one time point) were used. After filtering the data acquired from the experiment with a fully induced *E. coli *expression system consists of 733 genes and the data acquired from the process with limited induction consists of 429 genes.

For the functional relevance test another *E. coli *experiment was used where various mutants were investigated under oxygen deprivation [16]. The mutants were designed to monitor the response from *E. coli *during an oxygen shift in order to target the a priori most relevant part of the transcriptional network by using six strains with knockouts of key transcriptional regulators in the oxygen response. These experiments provide expression profiles for 4205 genes derived from the original data set downloaded from the Gene Expression Omnibus [17] with accession GDS680 by applying the altering steps described in [18].

### Functional grouping

Cluster analysis is used to find groups of co-regulated genes in the microarray data without prior knowledge about the gene functions. However, by clustering expression profiles of co-expressed genes groups of genes with similar function are often found.

The annotation of genes to categories or classes is a very important aspect in the analysis of gene expression data. The genes can for example be mapped to functional groups like Gene Ontology (GO, [9]) classifications or to protein complexes. Gene functions are very complex, therefore genes are usually mapped to multiple classes. In any case the mapping is known a priori and does not depend on the data of the currently investigated experiment.

External information about the annotation of genes to functional groups can easily be included in the neighborhood graph, e.g., the accumulation of gene ontology (GO) classifications in certain gene clusters can be highlighted in the node representation. In microarray data analysis gene ontology classifications about Biological Process, Molecular Function and Cellular Component are typically investigated. In this study experimental data from *E. coli *is used where further sources of external knowledge are the GenProtEC ([19], ) classification system for cellular and physiological roles of *E. coli *gene products and the RegulonDB ([20], ) for detailed information about operons and regulons.

### Software and implementation

All cluster algorithms and visualization methods used are implemented in the statistical computing environment R[21]. R package **flexclust **[7] is a flexible toolbox to investigate the influence of distance measures and cluster algorithms. It contains extensible implementations of the K-centroids and QT-Clust algorithm and offers the possibility to try out a variety of distance or similarity measures as cluster algorithms are treated separately from distance measures. New distance measures and centroid computations can easily be incorporated into cluster procedures. The default plotting method for cluster solutions in **flexclust **is the neighborhood graph.

A linear projection of the data into 2 dimensions using for example linear discriminant analysis (LDA) has the advantage that the lengths of edges in the graph are directly interpretable. However, LDA does not scale well in the number of clusters, and relationships between the centroids of more than 15 clusters can hardly be displayed in the plane. As shown in [22] linear methods cannot be used for high-dimensional gene expression data and a large number of clusters. R package **gcExplorer **[8] uses non-linear layout algorithms implemented in the open source graph visualization software Graphviz () for the display of neighborhood graphs. Bioconductor packages **graph **and **Rgraphviz **[23] provide tools for creating, manipulating, and visualizing graphs in R as well as an interface to Graphviz. **Rgraphviz **returns the layout information for a graph object, x- and y-coordinates of the graph's nodes as well as the parameterization of the trajectories of the edges. Several layout algorithms can be chosen:

**dot**: hierarchical layout algorithm for directed graphs 

**neato and fdp**: layout algorithms for large undirected graphs

**twopi**: radial layout

**circo**: circular layout

The default layout algorithm in **gcExplorer **is "dot". Even though distances between nodes and length of edges are no longer interpretable when using non-linear layout algorithms the increase in readability and clear arrangement is obvious.

The latest release of **gcExplorer **is always available at the Comprehensive R Archive Network CRAN: (). Details on how to use the **gcExplorer **can be found in the online appendix [see Additional file 1 for the vignette and Additional file 2 for the corresponding R code].

## Results and Discussion

### Exploratory analysis

Now the PS19 data is used to demonstrate the new functionality of **gcExplorer**. The data is clustered using stochastic QT-Clust [24] yielding a cluster object which consists of 14 clusters.

**Figure 1:**
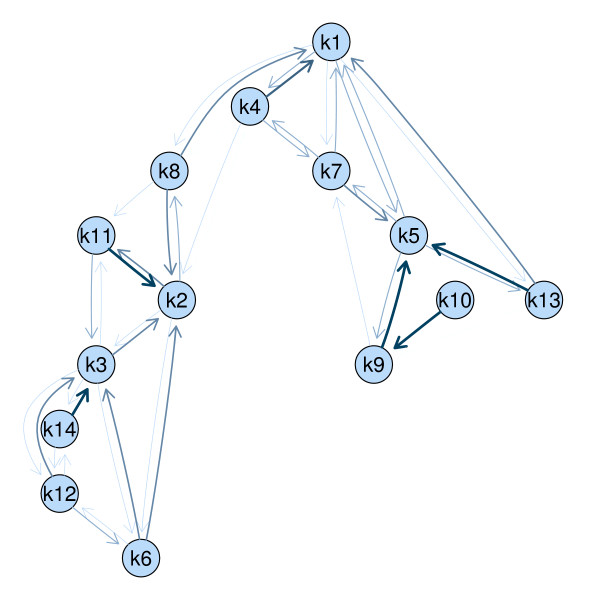
**Neighborhood graph**. Neighborhood graph of a cluster solution of the PS19 data.

The neighborhood graph of the cluster solution shown in Figure 1 allows a detailed view on the cluster structure even for a large number of clusters. The nodes in the graph correspond to cluster centroids and the shadow values between clusters defined above are used as edge weights. The thickness of an edge between two clusters is proportional to their similarity. Related clusters are not forced to lie next to each other in the graph as edges can have various lengths. For example cluster 13 located at the right end of the graph is related to cluster 1 located in the top of the graph. Several groups of clusters can be found. The clusters in the bottom left corner of the graph (e.g., clusters 3, 6, 12 and 14) are not connected to the clusters in the right part of the graph (e.g., clusters 5, 9, 10 and 13) indicating that the corresponding genes show very different expression profiles over time.

#### Color coding of nodes

In the graph shown above one single kind of node symbol is used for all nodes. This way no information about the different clusters is revealed. There are several possibilities how to include additional information in the representation of nodes. The most simple method is to use color coding, e.g., to color nodes by size or tightness of the corresponding clusters. In this case the color of a node depends on the distribution of a certain property over all nodes where the maximum will get the darkest and the minimum will get the brightest color. Usually the smaller or tighter clusters are more interesting and can more easily be explored. The percentage of genes in a cluster assigned to a functional group under investigation can also be used for color coding. The visualization of functional groups in the graph is not only a validation of the cluster method. It is also a very helpful tool for practitioners to quickly find subgroups of genes related to specific functions under study.

**Figure 2:**
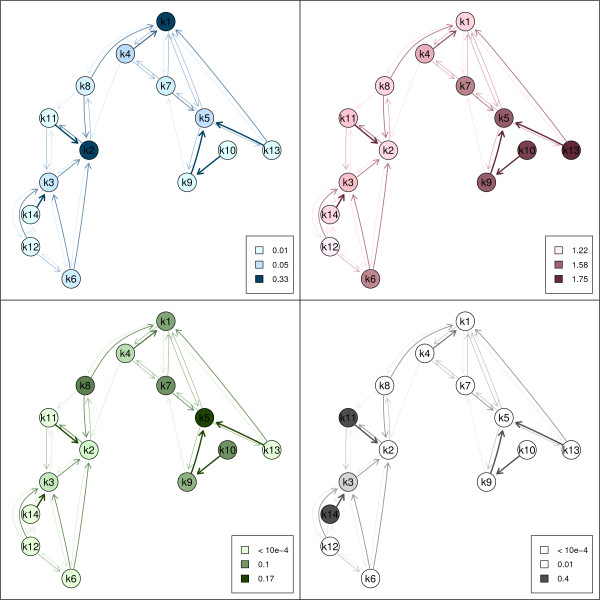
**Color coding**. Different options for color coding. Top left panel: cluster size, top right panel: cluster tightness, bottom left panel: Sigma 32 regulated genes, bottom right panel: genes involved in flagellar motility.

Some examples of color coding are shown in Figure 2. In panel (a) cluster size is highlighted, i.e., dark node symbols indicate large clusters and light node symbols indicate small clusters. In panel (b) cluster tightness is used where dark nodes correspond to tight clusters which usually correspond to groups of genes with clearly defined gene expression profiles. In panels (c) and (d) two functional groups are investigated. In panel (c) clusters with accumulation of *σ*_32_-regulated genes are highlighted which are related to heat shock.

In panel (d) the GO term "flagellar motility" is shown which is part of the biological process classification. Flagellar motility is an example of a functional group where the corresponding genes have similar expression profiles and are therefore grouped into similar clusters (i.e., clusters 11, 3 and 14) which are connected by edges in the neighborhood graph. In the case of *σ*_32_-regulated genes (panel (c)) there is no clear relationship between the cluster solution and the functional group as the corresponding genes are located in various clusters.

#### Node symbols

The second option for adding further information to the display of the neighborhood graph is to use different graphical symbols for the representation of nodes. For that purpose **gcExplorer **makes use of R package **symbols **([25], ). **symbols **is based on Grid[26], a very flexible graphics system for R. Grid features viewports, i.e., rectangular areas allowing the creation of plotting regions all over the R graphic device. Due to the layout algorithms used in the **gcExplorer **nodes remain quite large allowing large viewports for the visualization of nodes. Several grid-based functions are implemented in package **symbols **which can directly be used as node functions in the **gcExplorer**.

**Figure 3:**
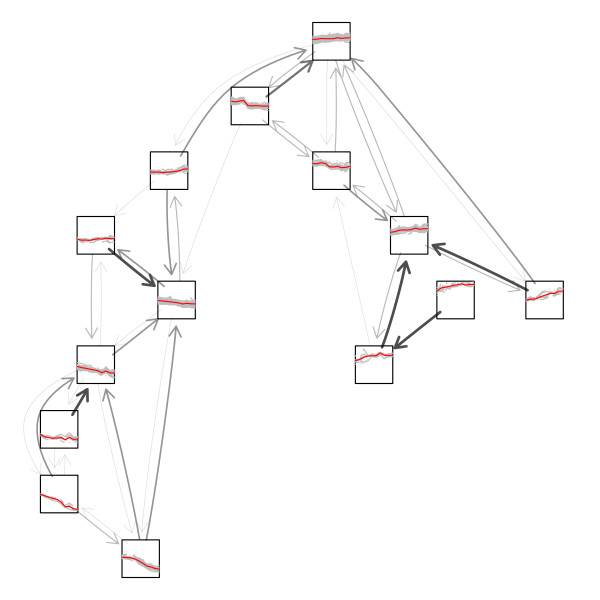
**Gene expression profiles as node symbols**. Neighborhood graph using line plots as node symbols where the gene expression profiles are plotted in grey and the cluster centroids are plotted in red.

The most natural node symbols in the case of time-course gene expression data are line plots showing the gene expression profiles over time for either the cluster centroids or the whole group of genes in a certain cluster. Figure 3 gives a very good overview of the cluster solution and the single gene clusters where similarities in gene expression profile can directly be investigated. It can be seen that clusters containing down-regulated genes are located in the bottom left part of the graph whereas up-regulated genes are located in the right part of the graph. Further, there are no edges between clusters of up- and down-regulated genes.

**Figure 4:**
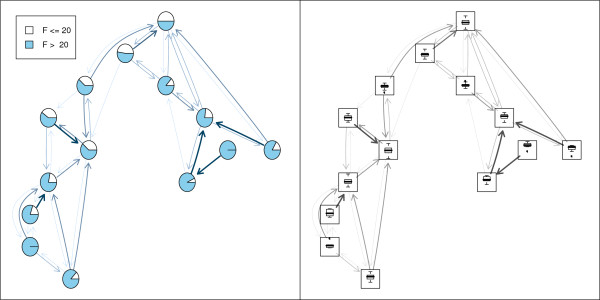
**Further node symbols**. Neighborhood graph using pie charts (left panel) and boxplots (right panel) as node symbols.

In order to visualize group memberships pie charts are frequently used. Figure 4 panel (a) shows the portion of genes with F statistic (F) > 20 and F ≤ 20 respectively. In panel (b) of Figure 4 boxplots of the log F statistic are shown.

#### Directed vs. undirected graph

The neighborhood graph is a directed graph as the similarity of cluster 1 to cluster 4 is different from the similarity of cluster 4 to cluster 1 and so on. Besides plotting the original directed graph there are several options how to plot edges taking into account for instance the mean, minimum or maximum of the similarities between two clusters. In practice the mean similarity is frequently used especially when testing the functional relationship between clusters (an example is given below).

#### Graph modifications

The non-linear layout algorithms implemented in Graphviz are optimized for the given set of nodes and edges. Removing an edge or a node will result in a different graph which makes comparisons between graphs rather complicated. R package **gcExplorer **contains the function gcModify which allows to modify a given graph without changing the original layout. There are several possibilities how to modify a given graph. However, it is only possible to remove nodes and edges from a larger graph. Adding new nodes and edges is not allowed. The node symbols are independent of the graph structure so different node functions can be used in each modified graph.

**Figure 5:**
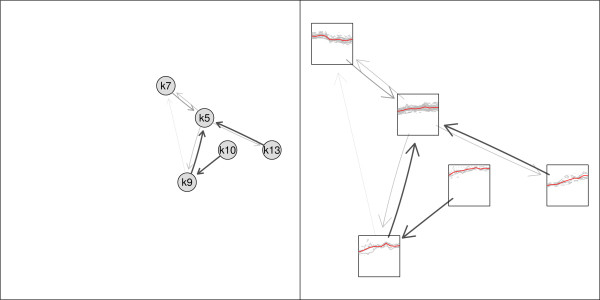
**Subgraph**. A subgraph of the neighborhood graph before zooming without specified node function (left panel) and after zooming with node function (right panel).

**Figure 6:**
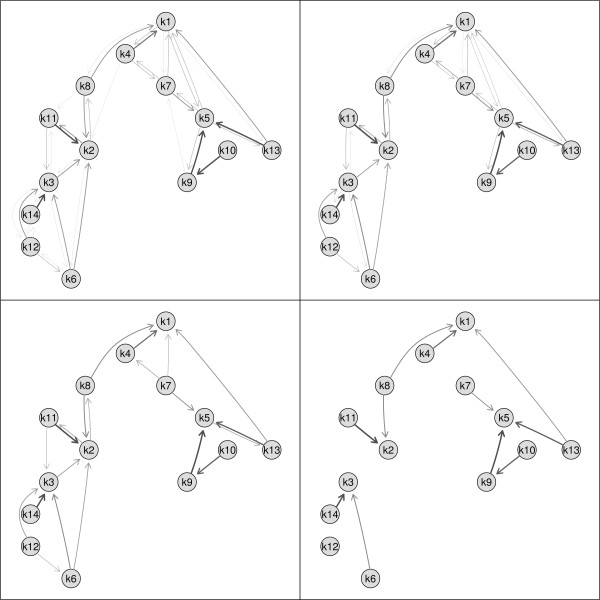
**Edge modifications**. Use of different cutoff values for drawing edges in the neighborhood graph. Top left panel: all edges, top right panel: similarity > 10%, bottom left panel: similarity > 20%, bottom right panel: similarity > 30%.

Sometimes only a subgraph of the original graph is of interest, e.g., clusters of all up-regulated genes. A subgraph can be created specifying either the set of nodes which should remain in the graph or by specifying the nodes which should be removed from the graph. In the next step manual or automatic zooming can be used to enlarge certain parts of the plot. An example of a subgraph is given in Figure 5. Filtering by cluster similarity can be used to simplify the original neighborhood graph. Edges between nodes are only drawn if the similarity between clusters is above a certain threshold, e.g., at least 10%. This prevents the graph from being too complex. Examples of the neighborhood graph where different cutoff values for drawing edges are shown are given in Figure 6.

Comparisons of different cutoff values as shown in Figure 6 are only possible when starting with the largest set of edges.

### Inferential analysis

#### Compare cluster solutions

Finally the goodness of the cluster solution of the PS19 data investigated so far is judged based on its validity when applied to the PS17 experiment where the same set of genes was exposed to different experimental conditions. Table 1 gives the results of the comp_test consisting of cluster size, observed average within cluster distance, the 5% quantile of the permuted average distances and the probability of observing a lower within cluster distance by randomly assigning the genes to clusters. In this case 10 out of 14 clusters have a significantly smaller within cluster distance when using the cluster solution of the PS19 experiment compared to random assignment. In other words these 10 groups of genes form clusters under different experimental conditions and are more likely to contain co-regulated genes.

**Table 1:** Result of comp_test. Judge the validity of the PS19 cluster solution for the PS17 data using the comp_test.

	**size**	**obs. av. dist**	**5% quantile. perm**	**p. val. lower**
1	302	0.58	0.95	**0.00**
2	299	0.55	0.94	**0.00**
3	41	0.65	0.83	**0.00**
4	59	0.62	0.85	**0.00**
5	52	0.73	0.84	**0.00**
6	31	0.61	0.79	**0.00**
7	30	0.66	0.78	**0.00**
8	26	0.82	0.77	0.10
9	14	0.52	0.68	**0.00**
10	10	0.38	0.62	**0.00**
11	10	0.70	0.63	0.12
12	5	0.49	0.45	0.07
13	12	0.96	0.66	0.53
14	10	0.62	0.63	**0.04**

#### Functional relevance test

**Figure 7:**
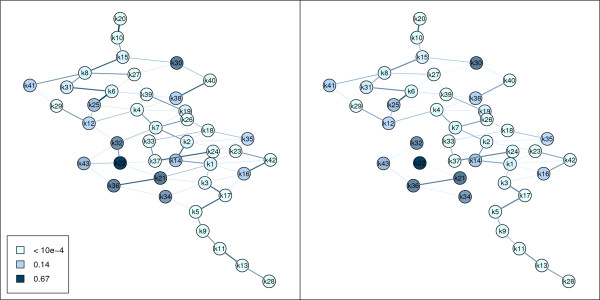
**Functional relevance rest**. Left Panel: Neighborhood graph of the oxygen data set where the mean edge method is used. Right Panel: Neighborhood graph where significant edges are removed using the functional relevance test.

Another possibility for external validation of a cluster solution is to test the functional relevance of single edges, i.e., to test the relationship between a functional grouping and a cluster solution. In this example the *E. coli *oxygen data set [16] is used and the GO term GO:0009061 (anaerobic respiration) is investigated. The accumulation of genes involved in anaerobic respiration is displayed in Figure 7 left panel. In the case of edge tests undirected graphs are used instead of the original directed graphs as each pair of nodes is only tested once.

The output of function edgeTest (see Table 2) gives detailed information about the tested edges, i.e., the corresponding cluster sizes, the difference in proportions and the p-value. Additionally, function edgeTest gives the 95% quantile of the maxima of the permuted average distances which is 0.22 in this case. The p-values are now used to form a new similarity matrix using function newclsim. If the p-value of an edge is smaller than 0.05 the edge weight is set to 0. This new similarity matrix based on the p-values of the functional relevance test is finally used to draw a modified neighborhood graph where significant edges are removed. In this case 11 edges have significant p-values and differences in proportions larger than 0.23. In Figure 7 right panel the modified neighborhood graph is displayed. It can be seen that clusters 32, 43, 36, 34, 21 and 22 contain most of the genes involved in anaerobic respiration and form a disconnected subgraph after testing the functional relevance of the edges.

**Table 2:** Result of functional relevance test.

	**Clsize1**	**Clsize2**	**Diff. in. Prop.**	**P-value**
1^~^2	671	526	0.02	1.00
1^~^3	671	424	0.01	1.00
4^~^6	378	209	0.02	1.00
2^~^7	526	121	0.01	1.00
4^~^7	378	121	0.02	1.00
6^~^8	209	108	0.01	1.00
4^~^12	378	16	0.11	0.59
1^~^14	671	33	0.14	0.51
2^~^14	526	33	0.16	0.50
1^~^16	671	13	0.11	0.59
3^~^16	424	13	0.12	0.57
1^~^21	671	9	0.40	**0.00**
3^~^21	424	9	0.41	**0.00**
14^~^21	33	9	0.26	**0.05**
14^~^22	33	12	0.48	**0.00**
21^~^22	9	12	0.22	0.13
4^~^25	378	10	0.19	0.29
6^~^25	209	10	0.17	0.34
12^~^25	16	10	0.08	0.93
2^~^32	526	11	0.34	**0.01**
7^~^32	121	11	0.33	**0.03**
12^~^32	16	11	0.24	**0.05**
22^~^32	12	11	0.30	**0.03**
3^~^34	424	6	0.30	**0.03**
5^~^34	263	6	0.33	**0.03**
21^~^34	9	6	0.11	0.77
2^~^35	526	17	0.09	0.81
21^~^36	9	5	0.04	1.00
34^~^36	6	5	0.07	0.94
22^~^43	12	9	0.44	**0.00**
32^~^43	11	9	0.14	0.51
36^~^43	5	9	0.18	0.33

Functional relevance test of the *E. coli *oxygen data for functional group GO:0009061 (anaerobic respiration).

#### Power simulations for the functional relevance test

The power of the functional relevance test is simulated on artificial cluster solutions. For defined

• datasize

• number of clusters

• difference in proportions between cluster 1 and 2

• proportion of grouped genes in cluster 1

• proportion of grouped genes in the total data set

a cluster solution is simulated where the difference in proportions between clusters 1 and 2 is fixed and the remaining proportions are random. For a given setup the functional relevance test is run 1000 times where only the power for the edge between clusters 1 and 2 is observed (see Table 3). The number of clusters is 10 in all data sets. It can be seen that the test performs best if the proportion of grouped genes in cluster 1 is large and the proportion of grouped genes in the total data set is small.

**Table 3:** Power simulations for the functional relevance test.

**Data size**	**prop. c1**	**prop. all**	**d 0.05**	**d 0.1**	**d 0.15**	**d 0.2**	**d 0.25**	**d 0.3**	**d 0.35**	**d 0.4**
100	0.50	0.50	0	0.000	0.000	0.000	0.004	0.043	0.062	0.108
100	0.50	0.33	0	0.000	0.000	0.000	0.010	0.044	0.095	0.179
100	0.50	0.25	0	0.000	0.000	0.000	0.011	0.074	0.129	0.229
100	0.50	0.20	0	0.000	0.000	0.001	0.018	0.078	0.186	0.300

100	0.33	0.50	0	0.000	0.001	0.005	0.033	0.051	0.033	0.029
100	0.33	0.33	0	0.000	0.000	0.006	0.035	0.068	0.071	0.044
100	0.33	0.25	0	0.000	0.000	0.013	0.049	0.065	0.074	0.062
100	0.33	0.20	0	0.000	0.001	0.020	0.064	0.087	0.088	0.080

500	0.50	0.50	0	0.000	0.010	0.084	0.276	0.653	0.999	1.000
500	0.50	0.33	0	0.000	0.015	0.137	0.442	0.918	1.000	1.000
500	0.50	0.25	0	0.000	0.010	0.180	0.606	0.996	1.000	1.000
500	0.50	0.20	0	0.000	0.025	0.248	0.700	1.000	1.000	1.000

500	0.33	0.50	0	0.001	0.026	0.159	0.384	0.747	0.764	0.450
500	0.33	0.33	0	0.001	0.069	0.242	0.551	0.978	0.889	0.669
500	0.33	0.25	0	0.002	0.074	0.301	0.733	1.000	0.909	0.905
500	0.33	0.20	0	0.000	0.098	0.414	0.903	1.000	0.935	0.976

Power simulations for the functional relevance test using differences in proportion between 0.05 and 0.4.

## Conclusion

Clustering gene expression profiles is a helpful tool for finding biologically meaningful groups of genes without prior information from databases. As the definition of gene clusters is not very clear and genetic interactions are extremely complex the relationship between clusters is very important and co-expressed genes can end up in different clusters. In order to make cluster analysis useful for practitioners the interactive visualization tool **gcExplorer **was developed. It allows not only to visualize the cluster structure in form of neighborhood graphs, beyond the gene clusters are plotted or shown in HTML tables with links to databases. In this paper recent extensions of the package were presented including different node representations using node coloring and the choice of node symbols. Additional properties of the clusters like cluster size or cluster tightness can be highlighted as well as external information like functional grouping. Graphs can be modified by removing nodes and edges or by zooming into a subgraph of interest. Further, the functional relevance of a clustering can be tested using external information about gene function from databases. Finally, the validity of a cluster solution can be judged based on its performance on another data set where the same set of genes is investigated under different experimental conditions.

## Availability and requirements

Project name: gcExplorer; Project home page: . Operating system(s): A wide variety of UNIX platforms, Windows and MacOS. Programming language: R; License: GPL-2.

The gcExplorer package and its associated packages are part of the R/Bioconductor project, an environment for statistical computing and bioinformatics. The R software environment is freely available at . The dependencies flexclust and Rgraphviz can be downloaded from CRAN  and the Bioconductor project website .

## Authors' contributions

TS implemented the software, carried out the analysis of the data and wrote the manuscript. IV contributed to the software. FL directed the research.

## Supplementary Material

Additional file 1 **gcExplorer Vignette**. A detailed description of how to perform the analysis with the gcExplorer shown in this paper. Click here for file

Additional file 2 **R Code**. The corresponding R commands to perform the analysis with the gcExplorer shown in this paper. Click here for file
