# Supplementary material for: Exploratory and inferential analysis of gene cluster neighborhood graphs
Source: BMC Bioinformatics. 2009 Sep 14;10:288. doi: 10.1186/1471-2105-10-288 (PMC2761902; doi:10.1186/1471-2105-10-288)
Supplement: Additional file 1 — gcExplorer Vignette. A detailed description of how to perform the analysis with the gcExplorer shown in this paper. [file 1471-2105-10-288-S1.pdf]

How to use the gcExplorer: Online Appendix to the Paper "Exploratory and Inferential Analysis of Gene Cluster Neighborhood Graphs"

Theresa Scharl Ingo Voglhuber Friedrich Leisch

July 30, 2009

Contents

1 Overview **1**

2 Exploratory Analysis **2**
2.1 Interactive exploration 2
2.2 Node Functions 5
2.2.1 Color coding 5
2.2.2 Node symbols 8
2.3 Graph Modifications 12
2.3.1 Node modifications 12
2.3.2 Edge modifications 13

3 Inferential Analysis **13**
3.1 Compare Cluster Solutions 13
3.2 Functional Relevance Test 15

4 Sessioninfo **17**

1 Overview

This document is an additional file to the paper "Exploratory and Inferential Analysis of Gene Cluster Neighborhood Graphs" by Scharl, Voglhuber and Leisch submitted to BMC Bioinformatics where recent extensions of *R* package *gcExplorer* (Scharl and Leisch, 2009) are presented. This document will also be contained in the package as a vignette. Here we give the *R* code for the analysis described in the paper. Details about the different options and arguments can be found in the paper and in the help pages of the functions. *gcExplorer* depends on *R* package *flexclust* (Leisch, 2006) and Bioconductor package *Rgraphviz* (Carey et al., 2005).

2 Exploratory Analysis

2.1 Interactive exploration

First the *E. coli* PS19 data is clustered using the stochastic QT–Clust algorithm implemented in function `qtclust` of package *flexclust*.

```
> library("gcExplorer")
> data("ps19")
> set.seed(1111)
> cl1 <- qtclust(ps19, radius = 2, save.data = TRUE,
+         control = list(min.size = 5))
> cl1

kccasimple object of family 'kmeans'

call:
qtclust(x = ps19, radius = 2, control = list(min.size = 5),
    save.data = TRUE)

cluster sizes:

   1    2    3    4    5    6    7    8    9   10
 302  299   41   59   52   31   30   26   14   10
  11   12   13   14 <NA>
  10    5   12   10   17
```

The resulting cluster object consisting of 14 clusters is visualized using *gcExplorer* (see Figure 1). A color theme can be specified using argument `theme`. Argument `filt` can be used to specify which edges should be plotted, i.e., two centroids are only connected in the graph if the similarity is above a certain threshold. Argument `layout` can be used to specify one of the non–linear layout algorithms implemented in *Rgraphviz*:

dot: hierarchical layout algorithm for directed graphs

neato and fdp: layout algorithms for large undirected graphs

twopi: radial layout

circo: circular layout

```
> gcExplorer(cl1, layout = "dot", theme = "blue", filt = 0)
```

The interactive `gcExplorer` can be called using an arbitrary panel function, e.g.,

```
> gcExplorer(cl1, theme = "blue", filt = 0,
+         panel.function = gcProfile)
```

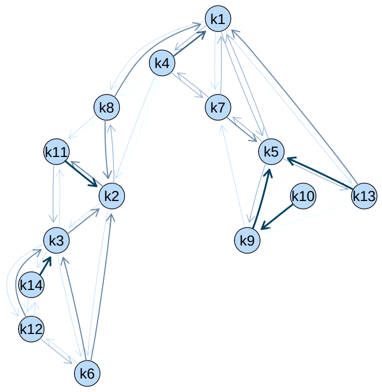

Figure 1: Neighborhood graph of a cluster solution of the PS19 data

for line plots showing the corresponding gene expression profiles. An example of the interactive usage of the *gcExplorer* is given in Figure 2. By clicking on the nodes of the neighborhood graph new graphics devices pop up showing the corresponding cluster by using the stated panel function. In this example clusters 3, 4, 7, 13 and 14 are visualized by plotting the corresponding gene expression profiles and cluster 3 is also displayed in form of an html table using panel function `gcTable`.

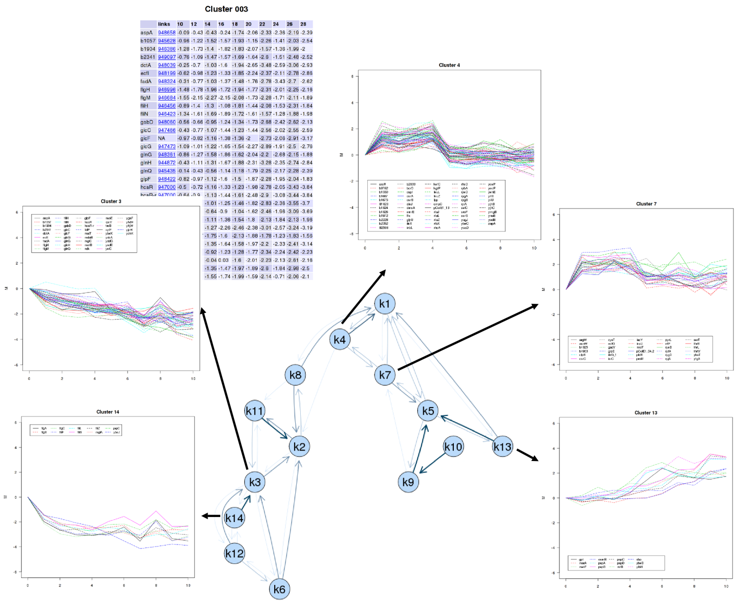

Figure 2: Neighborhood graph with some of the clusters displayed using panel functions `gcProfile` and `gcTable`.

2.2 Node Functions

2.2.1 Color coding

Further information can be added to the neighborhood graph by the use of color coding specified by argument `node.function`. Some examples of color coding are shown in Figure 3. The color theme can be modified using argument `theme`. In panel (a) cluster size is highlighted using function `node.size`, i.e., dark node symbols indicate large clusters and light node symbols indicate small clusters. A legend is added if the position of the legend is specified using argument `legend.pos`.

```
> gcExplorer(cl1, filt = 0, theme = "blue",
+         node.function = node.size,
+         legend.pos = "bottomright")
```

In panel (b) cluster tightness (node function `node.tight`) is used where dark nodes correspond to tight clusters which usually contain groups of genes with clearly defined gene expression profile.

```
> gcExplorer(cl1, filt = 0, theme = "red",
+         node.function = node.tight,
+         legend.pos = "bottomright")
```

In panels (c) and (d) two functional groups are investigated. In panel (c) clusters with accumulation of σ_{32}–regulated genes are highlighted which are related to heat shock response. The assignment of *E. coli* genes to Sigma factors is given in data `sigma`. In this case node function `node.go` is used where further arguments are passed using argument `node.args`. `gonr` is the name of the functional group under investigation, `source.id` and `source.group` contain gene identifiers and their assigned groups for the organism and `id` is the vector of identifiers for the clustered dataset.

```
> data("sigma")
> gcExplorer(cl1, filt = 0, theme = "green",
+         node.function = node.go,
+         node.args = list(gonr = "Sigma32",
+                          source.id = sigma[,4],
+                          source.group = sigma[,1],
+                          id = bn_ps19),
+         legend.pos = "bottomright")
```

In panel (d) the GO term "flagellar motility" which is part of the gene ontology biological process classification is shown. The assignment of *E. coli* genes to GO biological process terms is given in data `gobp`.

```
> data("gobp")
> gcExplorer(cl1, filt = 0,
+         node.function = node.go,
```

```
+         node.args = list(gonr = "flagellar motility",
+                          id = bn_ps19,
+                          source.group = gobp[,3],
+                          source.id = gobp[,1]),
+         legend.pos = "bottomright")
```

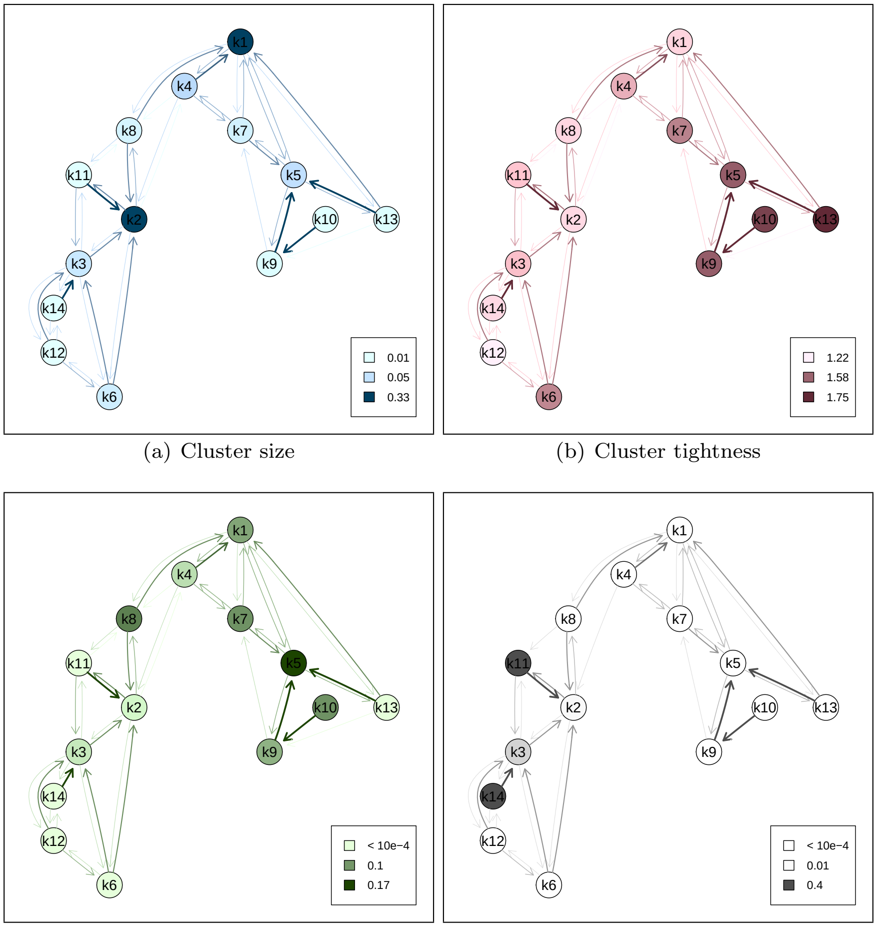

(a) Cluster size
(b) Cluster tightness
(c) Sigma 32
(d) Flagellar Motility

Figure 3: Different options for color coding

2.2.2 Node symbols

Another option for adding information to the display of the neighborhood graph is to use different graphical symbols for the representation of nodes. For that purpose *gcExplorer* makes use of *R* package *symbols* (`http://r-forge.r-project.org/projects/symbols`).

The most natural node symbols in the case of time–course gene expression data is to use line plots showing the gene expression profiles for either the cluster centroids or the whole group of genes in a certain cluster.

First, a grid–based `node.function` has to be defined, e.g.,

```
> gmatplot <- function (object, cluster, bgdata) {
+         grid.rect()
+         data <- object@data@get("designMatrix")
+         ylimits <- c(min(data, na.rm = TRUE), max(data, na.rm = TRUE))
+         index <- (object@cluster == cluster)
+         nodedata <- data[index,]
+         symb.matplot(1:ncol(nodedata), t(nodedata), type = "l",
+                 col = "gray", ylim = ylimits, pch = 1)
+         center <- object@centers[cluster,]
+         symb.matplot(1:ncol(object@centers), center, type = "l",
+                 col = "red", ylim = ylimits, pch = 1)
+ }
```

Now this node function is used in the `gcExplorer` by setting argument `doViewPort = TRUE` which enables the use of viewports.

```
> gcExplorer(cl1, filt = 0,
+         node.function = gmatplot,
+         doViewPort = TRUE)
```

Figure 4 gives a very good overview of the cluster solution and the single gene clusters where similarities in gene expression profile can directly be investigated. It can be seen that clusters containing down–regulated genes are located in the bottom left part of the graph whereas up–regulated genes are located in the right part of the graph. Further, there are no edges between clusters of up- and down–regulated genes.

Another example for node symbols are pie charts. Here is a user–defined grid pie function

```
> gpie <- function (object, cluster, bgdata) {
+         clusterindex <- object@cluster
+         clusterindex[is.na(clusterindex)] <- 0
+         index <- (clusterindex == cluster)
+         A2.cl <- bgdata[index,]
+         NOgroup <- length(A2.cl[A2.cl])
+         groupA <-length(A2.cl[!A2.cl])
+         symb.pie(c(NOgroup,groupA), labels = "",
```

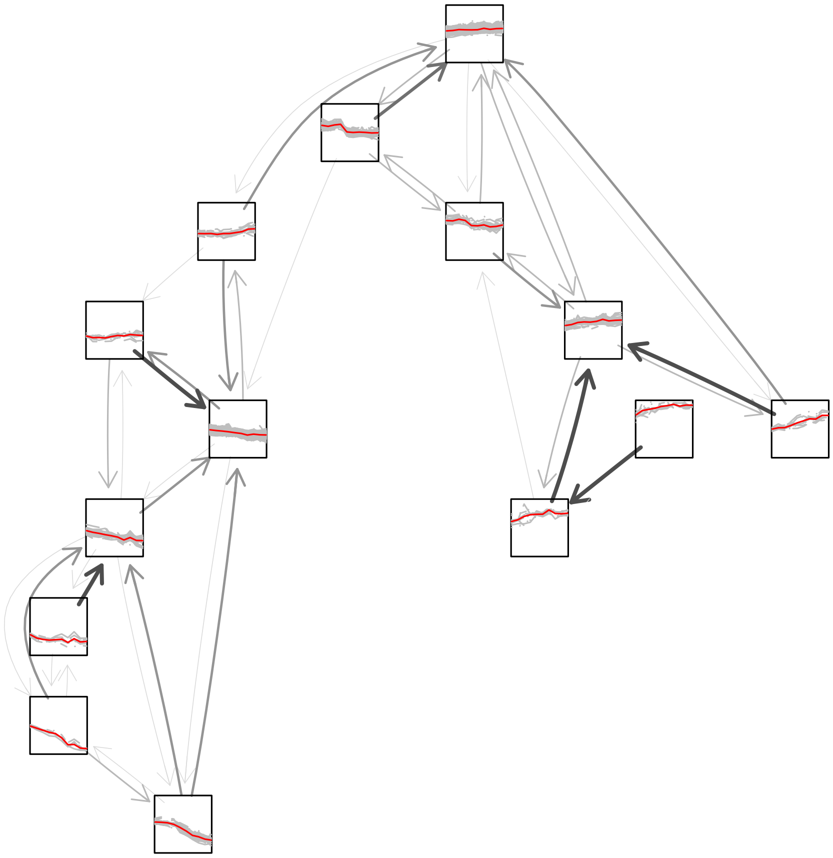

Figure 4: Neighborhood graph using line plots as node symbols where the genes expression profiles are plotted in grey and the cluster centroids are plotted in red.

```
+                 radius = 1.1,
+                 col = c("white", "skyblue"))
+ }
```

For demonstration purpose the F–statistic for differential expression for each gene is used here where the amount of genes with F–statistic ≤ 20 is given in white and the amount of genes with F–statistic > 20 is given in skyblue (see Figure 5 left panel.

```
> f2 <- f<20
> gcExplorer(cl1, filt = 0, theme = "blue",
+         node.function = gpie,
+         bgdata = as.data.frame(cbind(as.numeric(f2))),
+         doViewPort = TRUE)
> legend("topleft", inset = 0.05,
+         legend = c("F <= 20", "F >  20"),
+         fill = c("white", "skyblue"))
```

Grid–based boxplots can be used as node symbols using the following user–defined function.

```
> gbxp <- function (object, cluster, bgdata) {
+         ylim <- c(min(bgdata), max(bgdata))
+         index <- (object@cluster == cluster)
+         nodedata <- bgdata[index,]
+         symb.bxp(boxplot(nodedata, plot = FALSE),
+                 frame.plot = TRUE, ylim = ylim)
+ }
```

In the right panel of Figure 5 boxplots of the log F statistic are shown.

```
> gcExplorer(cl1, filt = 0,
+         node.function = gbxp,
+         bgdata = as.data.frame(log(f)),
+         doViewPort = TRUE)
```

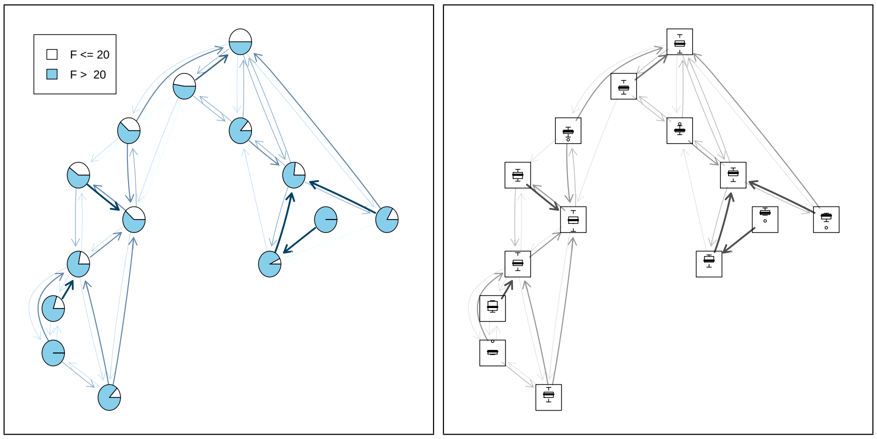

(a) Pie charts (b) Boxplots

Figure 5: Neighborhood graph using pie charts and boxplots.

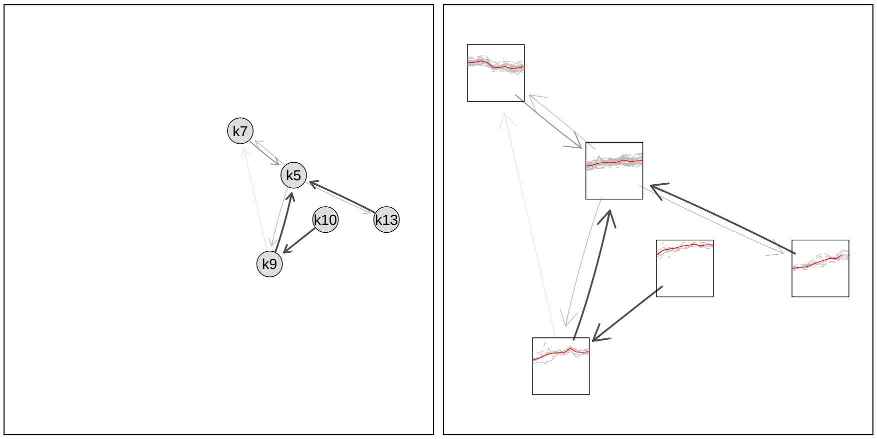

(a) Subgraph (b) Zoom

Figure 6: A subgraph of the neighborhood graph before zooming without specified node function (left panel) and after zooming with node function (right panel).

2.3 Graph Modifications

2.3.1 Node modifications

In order to modify an existing graph the graph structure has to be saved.

```
> graph <- gcExplorer(cl1, filt = 0,
+          node.function = gmatplot,
+          doViewPort = TRUE)
```

Now the graph structure of object `graph` can be modified using function `gcModify`. In this example argument `kpNodes` is used to keep only the stated nodes.

```
> graph1 <- gcModify(graph,
+          kpNodes = c("k5", "k7", "k9", "k10", "k13"),
+          doViewPort = FALSE)
```

The remaining subgraph is now investigated in detail using the `zoom` argument.

```
> graph2 <- gcModify(graph1, zoom = "auto")
```

In the left panel of Figure 6 the subgraph is shown with no node function setting argument `doViewPort=FALSE`. In the right panel the zoomed subgraph is shown.

2.3.2 Edge modifications

Filtering by cluster similarity can be used to simplify the original neighborhood graph. Edges between nodes are only drawn if the similarity of a cluster to another cluster is above a certain threshold, e.g., at least 10%. This prevents the graph from being too complex.

Now the similarity matrix is modified.

```
> d1 <- clusterSim(cl1)
> d1[d1 < 0.1] <- 0
> d2 <- d1
> d2[d2 < 0.2] <- 0
> d3 <- d2
> d3[d3 < 0.3] <- 0
```

Here `d1` is the original cluster similarity matrix which can be extracted from the cluster object using function `clusterSim`, `d2` is the similarity matrix where all values smaller 0.1 are set to 0 and so on.

Again we save the original neighborhood graph to object `graph`. In order to modify the edges of an existing graph function `gcModify` is used specifying argument `clsim`.

```
> graph <- gcExplorer(cl1, filt = 0)
```

```
> gcModify(graph, clsim = d1)
```

```
> gcModify(graph, clsim = d2)
```

```
> gcModify(graph, clsim = d3)
```

Examples of the neighborhood graph where the different cutoff values for drawing edges are shown are given in Figure 7.

3 Inferential Analysis

3.1 Compare Cluster Solutions

Function `comp_test` is now used to test the goodness of the cluster solution obtained for the PS19 data when applied to the PS17 data where the same set of genes was investigated under different experimental conditions.

```
> data(comp19)
> ct1 <- comp_test(comp17, clusters(cl1), N = 1000)
```

The result is shown in Table 1 consisting of cluster size, observed average within cluster distance, the 5% quantile of the permuted average distances and the probability of observing a lower within cluster distance ("p.val.lower") by randomly assigning the genes to clusters. In this case 10 out of 14 clusters have a significantly smaller within cluster distance when using the cluster solution of

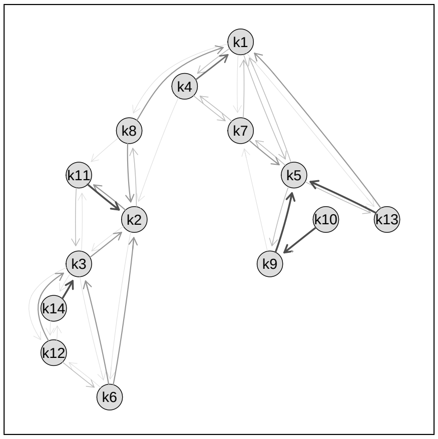

(a) All edges

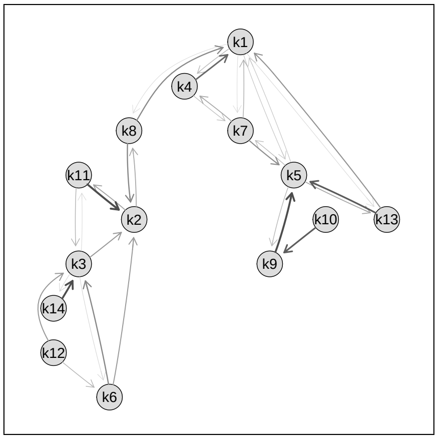

(b) Similarity > 10%

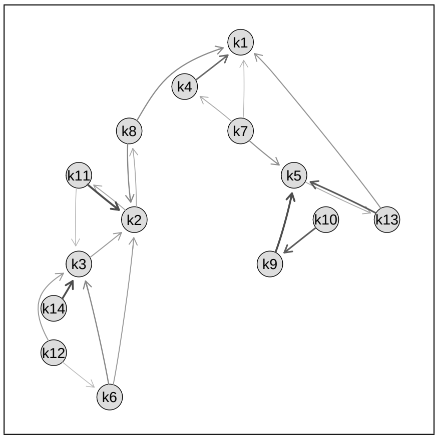

(c) Similarity > 20%

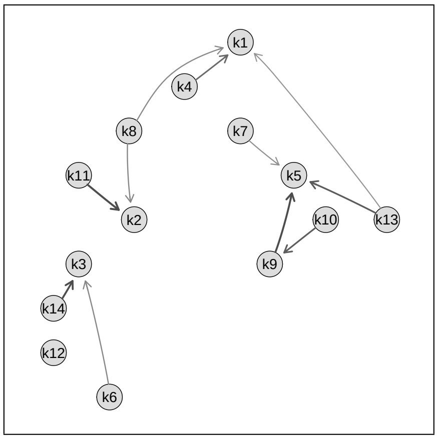

(d) Similarity > 30%

Figure 7: Use of different cutoff values for drawing edges in the neighborhood graph.

the PS19 experiment compared to random assignment. These 10 groups of genes form tight clusters under both conditions and therefore likely to be co–regulated.

	size	obs.av.dist	5%quantile.perm	p.val.lower
1	302.00	0.58	0.95	0.00
2	299.00	0.55	0.94	0.00
3	41.00	0.65	0.83	0.00
4	59.00	0.62	0.85	0.00
5	52.00	0.73	0.84	0.00
6	31.00	0.61	0.79	0.00
7	30.00	0.66	0.78	0.00
8	26.00	0.82	0.77	0.10
9	14.00	0.52	0.68	0.00
10	10.00	0.38	0.62	0.00
11	10.00	0.70	0.63	0.12
12	5.00	0.49	0.45	0.07
13	12.00	0.96	0.66	0.53
14	10.00	0.62	0.63	0.04

Table 1: Judge the validity of the PS19 cluster solution for the PS17 data using the comptest

3.2 Functional Relevance Test

Another possibility for external validation of a cluster solution is to test the functional relevance of single edges, i.e., to test the relationship between a functional grouping and a cluster solution. In this example the *E. coli* oxygen dataset Covert et al. (2004) is used and the GO term GO:0009061 (anaerobic respiration) is investigated.

The dataset is loaded and clustered into 43 clusters using `qtclust`.

```
> data(oxygen)
> set.seed(1111)
> cl2 <- qtclust(oxygen, radius = 3, save.data = TRUE,
+         control = list(min.size = 5))
> cl2

kccasimple object of family 'kmeans'

call:
qtclust(x = oxygen, radius = 3, control = list(min.size = 5),
    save.data = TRUE)

3288 points in 43 clusters, 100 outliers
```

```
Distribution of cluster sizes:
   Min. 1st Qu.  Median    Mean 3rd Qu.    Max.
   5.00    8.00   13.00   76.47   37.50  671.00
```

Function `Group2Cluster` is used to find the cluster membership of all genes involved in anaerobic respiration and the functional relevance test is implemented in function `edgeTest`. An edge is only tested if the number of functionally related genes is above a predefined threshold given by argument `min.size`. Argument `filt` can be used to filter edges with smaller than a predefined similarity threshold.

```
> g1 <- Group2Cluster(cl2, gonr = "GO:0009061",
+         source.group = gobp[,3], source.id = gobp[,1],
+         id = bn_oxy)
> eT <- edgeTest(cl2, group = g1, min.size = 2, filt = 0, N = 1000)
```

The output of function `edgeTest` (see Table 2 gives detailed information about the tested edges, i.e., the corresponding cluster sizes, the difference in proportions and the p–value. The 95% quantile of the maxima of the permuted average distances is 0.22 and can be extracted by

```
> eT$quant
```

The accumulation of genes involved in anaerobic respiration is displayed in Figure 8 left panel. Here `edge.method = "mean"` is used to draw an undirected graph. In this case a different layout algorithms is selected using `layout = "neato"`.

```
> graph <- gcExplorer(cl2, filt = 0, theme = "blue",
+         node.function = node.group,
+         node.args = list(group = g1),
+         layout = "neato",
+         edge.method = "mean",
+         legend.pos = "bottomleft")
```

The p-values are now used to form a new similarity matrix using function `newclsim`. If the p–value of an edge is smaller than 0.05 the similarity value is set to 0.

```
> clsim1 <- newclsim(eT = eT$res, object = cl2, p.filt = 0.05)
> gcModify(graph, clsim1)
```

In Figure 8 right panel the modified neighborhood graph is displayed. It can be seen that clusters 32, 43, 36, 34, 21 and 22 contain most of the genes involved in anaerobic respiration and form a disconnected subgraph after testing the functional relevance of the edges.

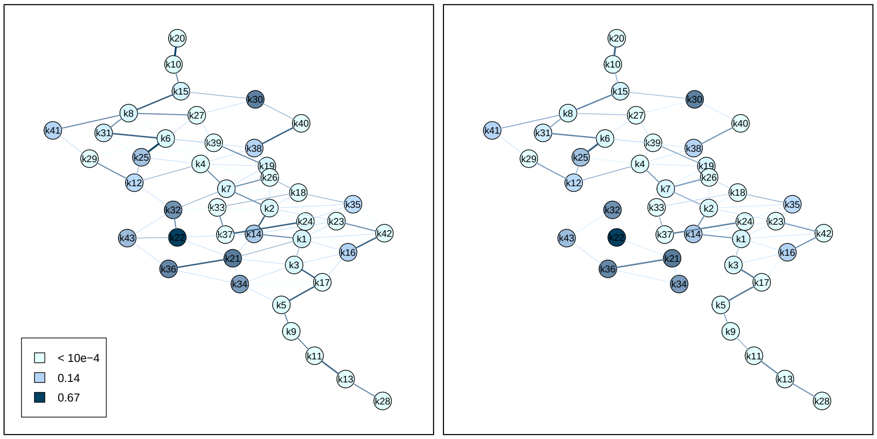

(a) All edges

(b) p-value > 0.05

Figure 8: Neighborhood graph using mean similarity values (left panel) and p–values of the functional relevance test (right panel) as edge weights.

4 Sessioninfo

This document was produced using

- R version 2.9.1 (2009-06-26), `x86_64-pc-linux-gnu`
- Locale: `LC_CTYPE=en_US;LC_NUMERIC=C;LC_TIME=en_US;LC_COLLATE=en_US;LC_MONETARY=C;LC_MESS`
- Base packages: base, datasets, graphics, grDevices, grid, methods, stats, stats4, utils
- Other packages: flexclust 1.2-1.1, gcExplorer 1.0-0, graph 1.22.2, lattice 0.16-2, modeltools 0.2-16, Rgraphviz 1.22.1, symbols 0.13, xtable 1.5-5
- Loaded via a namespace (and not attached): tools 2.9.1

together with version list(c(2, 20, 2)) of graphviz.

References

V. J. Carey, R. Gentleman, W. Huber, and J. Gentry. Bioconductor software for graphs. In R. Gentleman, V. J. Carey, W. Huber, R. A. Irizarry, and S. Dudoit, editors, *Bioinformatics and Computational Biology Solutions Using R and Bioconductor*, Statistics for Biology and Health. Springer-Verlag, New York, 2005. ISBN 978-0-387-25146-2.

M. Covert, E. Knight, J. Reed, M. Herrgard, and B. Palsson. Integrating high-throughput and computational data elucidates bacterial networks. *Nature*, 429(6987):92–96, 2004.

F. Leisch. A toolbox for k-centroids cluster analysis. *Computational Statistics and Data Analysis*, 51(2):526–544, 2006. doi: 10.1016/j.csda.2005.10.006.

T. Scharl and F. Leisch. gcexplorer: Interactive exploration of gene clusters. *Bioinformatics*, 25(8):1089–1090, 2009. doi: 10.1093/bioinformatics/btp099.

	Clsize1	Clsize2	Diff.in.Prop.	P-value
1˜2	671.00	526.00	0.02	1.00
1˜3	671.00	424.00	0.01	1.00
4˜6	378.00	209.00	0.02	1.00
2˜7	526.00	121.00	0.01	1.00
4˜7	378.00	121.00	0.02	1.00
6˜8	209.00	108.00	0.01	1.00
4˜12	378.00	16.00	0.11	0.59
1˜14	671.00	33.00	0.14	0.51
2˜14	526.00	33.00	0.16	0.50
1˜16	671.00	13.00	0.11	0.59
3˜16	424.00	13.00	0.12	0.57
1˜21	671.00	9.00	0.40	0.00
3˜21	424.00	9.00	0.41	0.00
14˜21	33.00	9.00	0.26	0.05
14˜22	33.00	12.00	0.48	0.00
21˜22	9.00	12.00	0.22	0.13
4˜25	378.00	10.00	0.19	0.29
6˜25	209.00	10.00	0.17	0.34
12˜25	16.00	10.00	0.08	0.93
2˜32	526.00	11.00	0.34	0.01
7˜32	121.00	11.00	0.33	0.03
12˜32	16.00	11.00	0.24	0.05
22˜32	12.00	11.00	0.30	0.03
3˜34	424.00	6.00	0.30	0.03
5˜34	263.00	6.00	0.33	0.03
21˜34	9.00	6.00	0.11	0.77
2˜35	526.00	17.00	0.09	0.81
21˜36	9.00	5.00	0.04	1.00
34˜36	6.00	5.00	0.07	0.94
22˜43	12.00	9.00	0.44	0.00
32˜43	11.00	9.00	0.14	0.51
36˜43	5.00	9.00	0.18	0.33

Table 2: Functional relevance test of the E. coli oxygen data for functional group GO:0009061 (anaerobic respiration)
